# Development of a prognostic nomogram for patients with malignant mesothelioma with bone metastasis

**DOI:** 10.1038/s41598-023-37679-9

**Published:** 2023-07-04

**Authors:** Awen Yang, Bin Tang, Xuan Liu, Jingxuan He, Qun Yan, Xianghui Liang, Wenen Liu

**Affiliations:** Department of Clinical Laboratory, Xiangya Hospital, Central South University, No.87 Xiangya Road, Kaifu District, Changsha, 410008 Hunan China

**Keywords:** Mesothelioma, Risk factors

## Abstract

Malignant mesothelioma (MM) is a rare aggressive tumor, and bone metastasis often occurs in later stages of this disease. This study aimed to establish a nomogram to predict the prognosis of bone metastasis of patients with MM. Data from the Surveillance, Epidemiology, and End Results database were screened and retrieved. This study included 311 patients with MM with bone metastases. Prognostic factors were analyzed using the Kaplan–Meier method and Cox proportional hazards model. A nomogram for overall survival (OS) was established and evaluated using statistically significant prognostic factors, and cancer-specific survival (CSS) analysis was performed to investigate its prognostic factors. In addition, the metastasis patterns of patients with MM were investigated, and the effects of different sites of metastasis on survival were compared using the Kaplan–Meier method. Age, sex, histological type, and chemotherapy were identified as the independent risk factors for OS. The 1-, 2-, and 3-year areas under the curve of the nomogram were 0.792, 0.774, and 0.928, and 0.742, 0.733, and 0.733 in the training and validation sets, respectively. Compared to OS, histological type, radiotherapy, and chemotherapy were independent risk factors for CSS. Different metastatic sites in MM have significantly different effects on prognosis.

## Introduction

Malignant mesothelioma (MM) is a rare aggressive tumor that mostly originates in the pleura (65–70%) and peritoneum (30%), and less commonly in the tunica vaginalis testis and pericardium (1–2%)^1^. The tunica of the testis and ovary is rarely affected^2^. MM is highly correlated with asbestos exposure^3^. According to a systematic analysis of the 2019 Global Burden of Disease Study, across 204 countries and territories, the age-standardized incidence rate of MM reached 0.4 (95% UI, 0.4–0.5 million) in 2019^4^. Despite the decrease in the figure in the past decades due to restrictions on asbestos exposure, it continues to increase in many territories^5^. Bone metastasis often occurs in the later stages of MM, and distant metastasis of tumors is often associated with worse prognosis. Therefore, it is important to study the prognosis of bone metastases in patients with MM in the clinical decision-making process.

Previous studies have suggested that MM has a wide range of prognostic factors. Sex, histological type, and treatment, affect the survival of patients^1^. However, most studies have focused on malignant pleural mesothelioma (MPM), a subtype of MM. Except for MPM, there is no definitive treatment method for other types of MM, so it is necessary to consider other types of MM for research^6^. Studies using nomograms for bone metastasis in patients with MM based on population-based data have not been reported. Therefore, we developed a nomogram using the Surveillance, Epidemiology, and End Results (SEER) database. Our findings may provide a batter understanding of patients with MM with bone metastases. In addition, no previous study has explored the metastatic patterns of MM in detail. In this study, the number of distant metastases of MM at each site was counted, and Kaplan–Meier analysis was used to evaluate the difference in the prognosis of MM according to different metastatic sites.

## Methods

### Data sources

The SEER database is a publicly available U.S. government-funded database that focuses on clinical information on patients with cancer^7^, (accession number: 12916-Nov2021), using SEER*Stat 8.4.0. Data were collected from patients with MM with bone metastases between 2000 and 2019.

### Participants

The primary cohort was selected as follows: (1) all cases identified as MM with ICD-O-3 histological type codes 9050/3, 9051/3, 9052/3, 9053/3, and 9055/3 and (2) all MM cases with bone metastases.

The exclusion criteria for the primary cohort were as follows: (1) cases without microscopic confirmation of an MM diagnosis and (2) cases with unknown clinical variables included in this study.

In addition, to investigate the effects of metastatic sites other than bone metastases on overall survival (OS), a data set including other metastatic sites was also included. The secondary cohort was selected as follows: all cases identified as MM with ICD-O-3 histological type codes 9050/3, 9051/3, 9052/3, 9053/3, and 9055/3.

The exclusion criteria for the secondary cohort were as follows: (1) cases without microscopic confirmation of an MM diagnosis and (2) cases with unknown metastatic sites.

### Clinical variables of malignant mesothelioma (MM) with bone metastasis

Clinical variables included demographic (age, sex, race, marital status, median household income, rural–urban continuum code), tumor-related (diagnostic confirmation, primary site, histological type), therapy-related (surgery of primary site, scope of regional lymph node surgery, radiotherapy recode, chemotherapy recode), and survival-related (survival months, vital status recode, SEER’s other cause of death classification) information. There were 13 variables in the final survival analysis.

In the SEER database, distant metastasis sites include the bone, brain, liver, and lung. Distant metastases were classified into 15 groups according to the different metastatic sites: four groups of single-organ, six groups of two-organ, four groups of three-organ, and one group of four-organ metastases.

### Data preprocessing

Using R version 4.1.2 (R Core Team, 2021) to preprocess the data with 344 samples, some cases were removed based on the exclusion criteria. The case selection flowchart is shown in Supplementary Figure S1. Age and survival information were imported into the X-tile software version 3.6.1 to obtain the best cut-off value and converted into ordinal categorical variables. The results in Supplementary Figure S2 show that the optimal cut-off values for age were 65 and 82 years.

### Statistical analyses

OS is defined as the time from diagnosis to death. For OS, univariate Cox regression analysis was performed on the 13 extracted variables. Then statistically significant predictors from the univariate analysis were included in the multivariate Cox regression, and variables with two-tailed p < 0.05 were considered statistically significant. The hazard ratios (HRs) and 95% confidence intervals (CIs) were calculated. For independent risk factors affecting survival, survival curves were drawn using the Kaplan–Meier method to determine the relationship between independent risk factors and survival, and the log-rank test method was applied for comparative analysis. The same analysis was performed for cancer-specific survival (CSS), which was measured as the time from diagnosis to death due to MM.

### Establishment and evaluation of the nomogram

After data preprocessing, 311 cases were divided into a 70% training set and a 30% validation set. The 1-, 2‐, and 3‐year OS probabilities were estimated using a nomogram based on the results of the independent risk factors. The model was evaluated using the receiver operating characteristic (ROC) curve. All statistical analyses were performed using R version 4.1.2 (R Core Team, 2021).

### Ethical approval

The data used in this study are publicly available and do not require approval from the ethics committee. All methods were carried out in accordance with relevant guidelines and regulations.

## Results

### Clinical baseline characteristics of patients with MM with bone metastases

Data from 344 patients diagnosed with MM with bone metastases were collected from the SEER database. After excluding patients whose clinical variables were unknown, 311 patients were included in the data analysis. Table 1 summarizes the baseline characteristics of 311 patients with MM with bone metastases. Among the included patients with MM with bone metastases, compared to females, males accounted for a larger proportion (80.71%), white people accounted for the largest proportion (87.14%), and patients’ age was predominantly between 65 and 82 years old (56.91%). More than half of the patients had a median household income of more than $60,000. The primary site in most patients was the pleura (86.5%). Among the histological types, sarcomatoid mesothelioma accounted for the largest proportion (26.69%), 10.93% underwent surgery at the primary site, 5.14% underwent lymphadenectomy, 95 (30.55%) underwent radiotherapy, and 150 (48.23%) received chemotherapy.

**Table 1:** Characteristics of patients.

Variables	Training set (*N*, %)	Validation set (*N*, %)	ALL (*N*, %)
Number of patients	217(70)	94(30)	311
Cause of death			
Alive or dead of other cause	28 (12.90)	11 (11.70)	39 (12.54)
Dead attributable to this cancer	189 (87.10)	83 (88.30)	272 (87.46)
Sex			
Female	47 (21.66)	13 (13.83)	60 (19.29)
Male	170 (78.34)	81 (86.17)	251 (80.71)
Race			
Black	10 (4.61)	3 (3.19)	13 (4.18)
White	189 (87.10)	82 (87.23)	271 (87.14)
Others	18 (8.29)	9 (9.57)	27 (8.68)
Age (years)			
≤ 65	61 (28.11)	21 (22.34)	82 (26.37)
66–82	122 (56.22)	55 (58.51)	177 (56.91)
> 82	34 (15.67)	18 (19.15)	52 (16.72)
Marital status			
Married	140 (64.52)	59 (62.77)	199 (63.99)
Divorced	18 (8.29)	9 (9.57)	27 (8.68)
Others	59 (27.19)	26 (27.66)	85 (27.33)
Median income			
< $39,999	10 (4.61)	1 (1.06)	11 (3.54)
$40,000–$49,999	26 (11.98)	9 (9.57)	35 (11.25)
$50,000–$59,999	37 (17.05)	16 (17.02)	53 (17.04)
$60,000–$69,999	64 (29.49)	31 (32.98)	95 (30.55)
> $70,000	80 (36.87)	37 (39.36)	117 (37.62)
Rural–urban continuum code			
Nonmetropolitan areas	27 (12.44)	7 (7.45)	34 (10.93)
Metropolitan areas	190 (87.56)	87 (92.55)	277 (89.07)
Primary site			
Pleura	190 (87.56%)	79 (84.04%)	269 (86.50%)
Peritoneum	4 (1.84%)	2 (2.13%)	6 (1.93%)
Others	23 (10.60%)	13 (13.83%)	36 (11.58%)
Histologict type			
Not otherwise specified	98 (45.16)	32 (34.04)	130 (41.80)
Sarcomatoid	55 (25.35)	28 (29.79)	83 (26.69)
Epithelioid	51 (23.50)	26 (27.66)	77 (24.76)
Biphasic	13 (5.99)	8 (8.51)	21 (6.75)
Surgery of primary site			
None	197 (90.78)	80 (85.11)	277 (89.07)
Resection	20 (9.22)	14 (14.89)	34 (10.93)
Scope of regional lymph node surgery			
None	208 (95.85)	87 (92.55)	295 (94.86)
Resection	9 (4.15)	7 (7.45)	16 (5.14)
Radiotherapy			
None/unknown	153 (70.51)	63 (67.02)	216 (69.45)
Yes	64 (29.49)	31 (32.98)	95 (30.55)
Chemotherapy			
None/unknown	119 (54.84)	42 (44.68)	161 (51.77)
Yes	98 (45.16)	52 (55.32)	150 (48.23)

### Survival analysis

**Figure 1:**
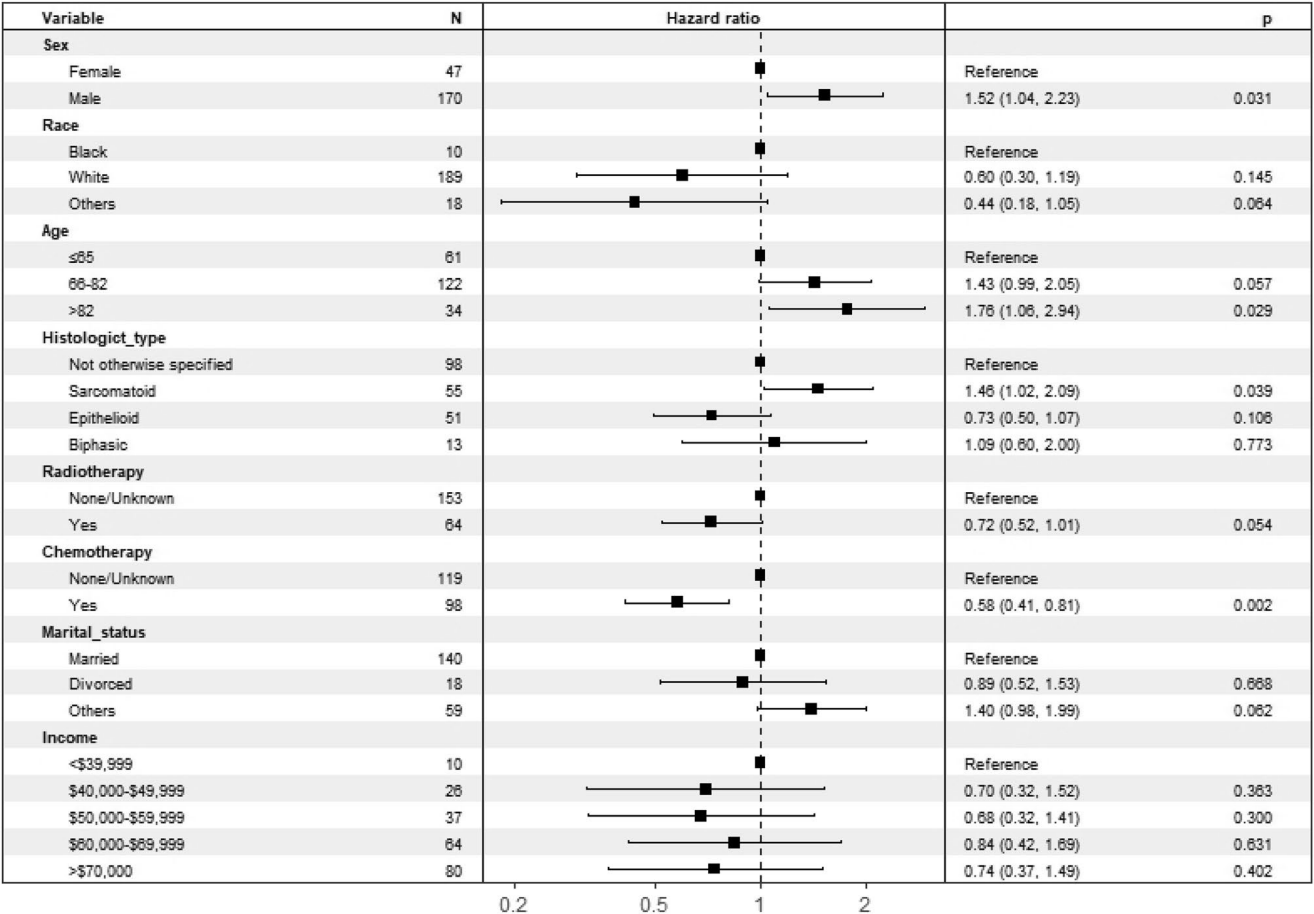
Multivariate analysis of overall survival (OS) in the OS training set.

**Figure 2:**
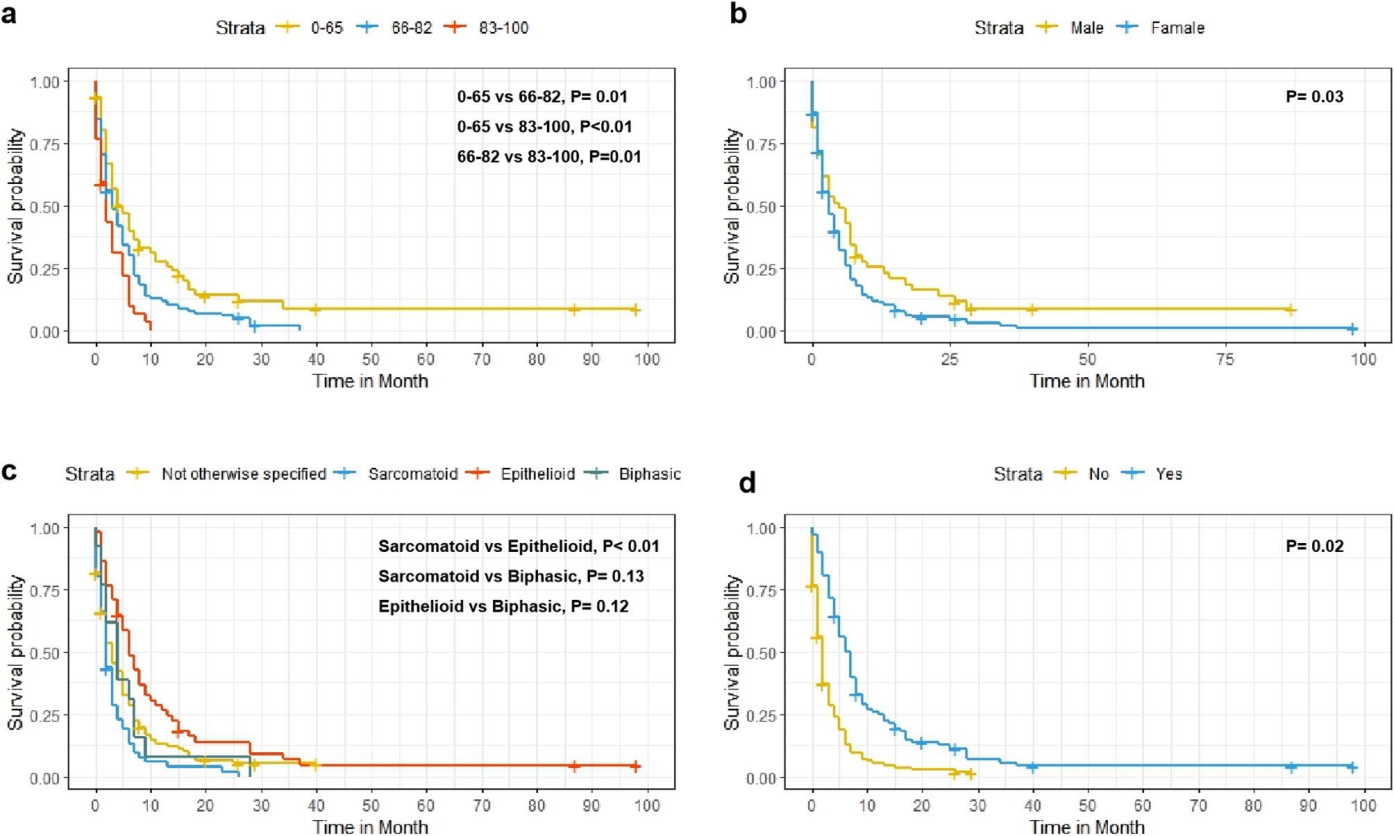
Predicted probability of overall survival by (**a**) age; (**b**) sex; (**c**) histologict type; (**d**) chemotherapy shown using Kaplan–Meier curve.

Variables found to be significantly associated with OS were sex, age, race, marital status, income, histological type, radiotherapy, and chemotherapy. After incorporating these variables into the multivariate analysis, the results showed that sex, age, histological type, and chemotherapy were independent risk factors for OS (Fig. 1). Combined with the results of the Kaplan–Meier survival analysis (Fig. 2), the above four independent risk factors were significantly associated with prognosis (p < 0.05).

**Figure 3:**
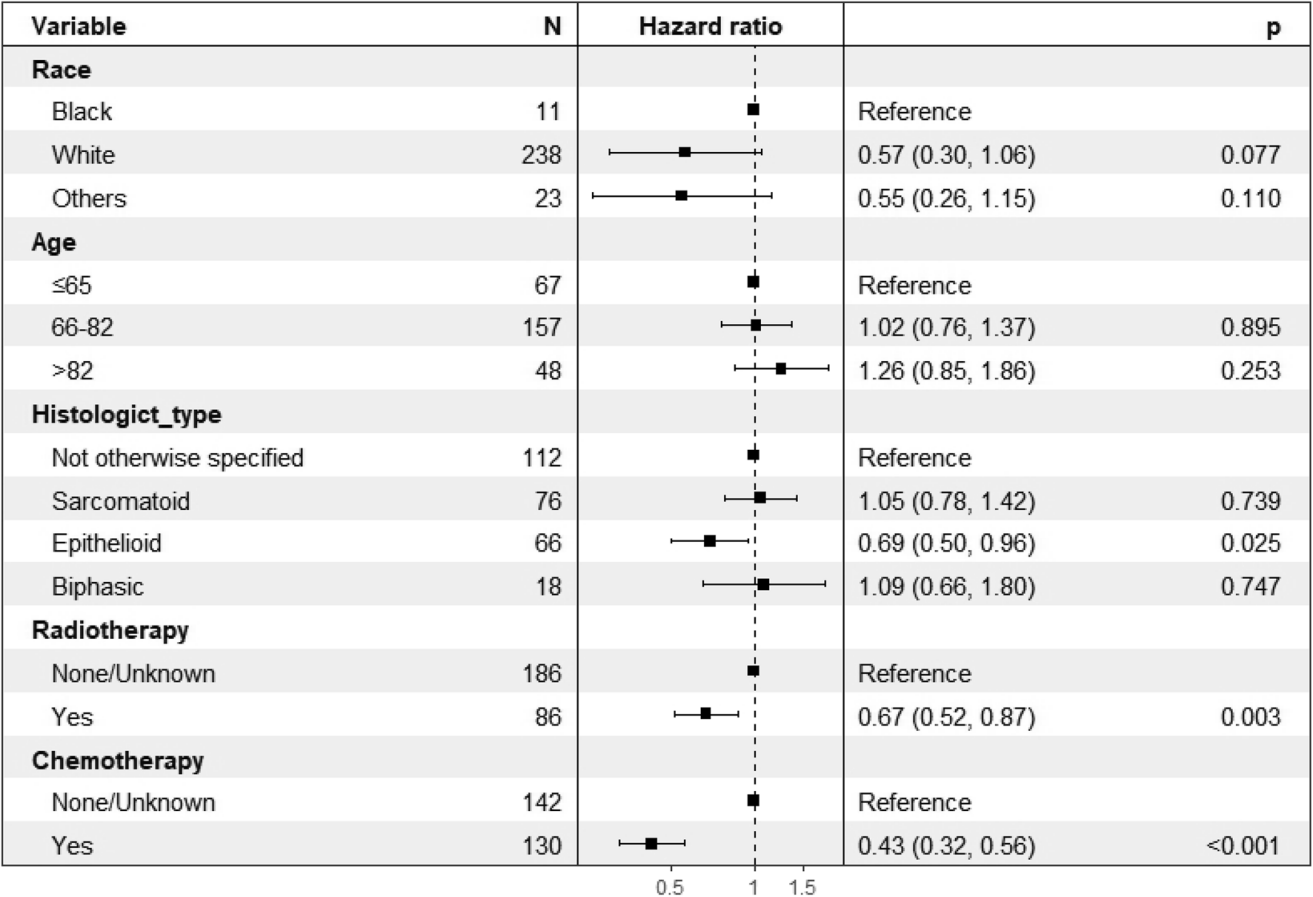
Multivariate analysis of cancer-specific survival (CSS) in the CSS dataset.

**Figure 4:**
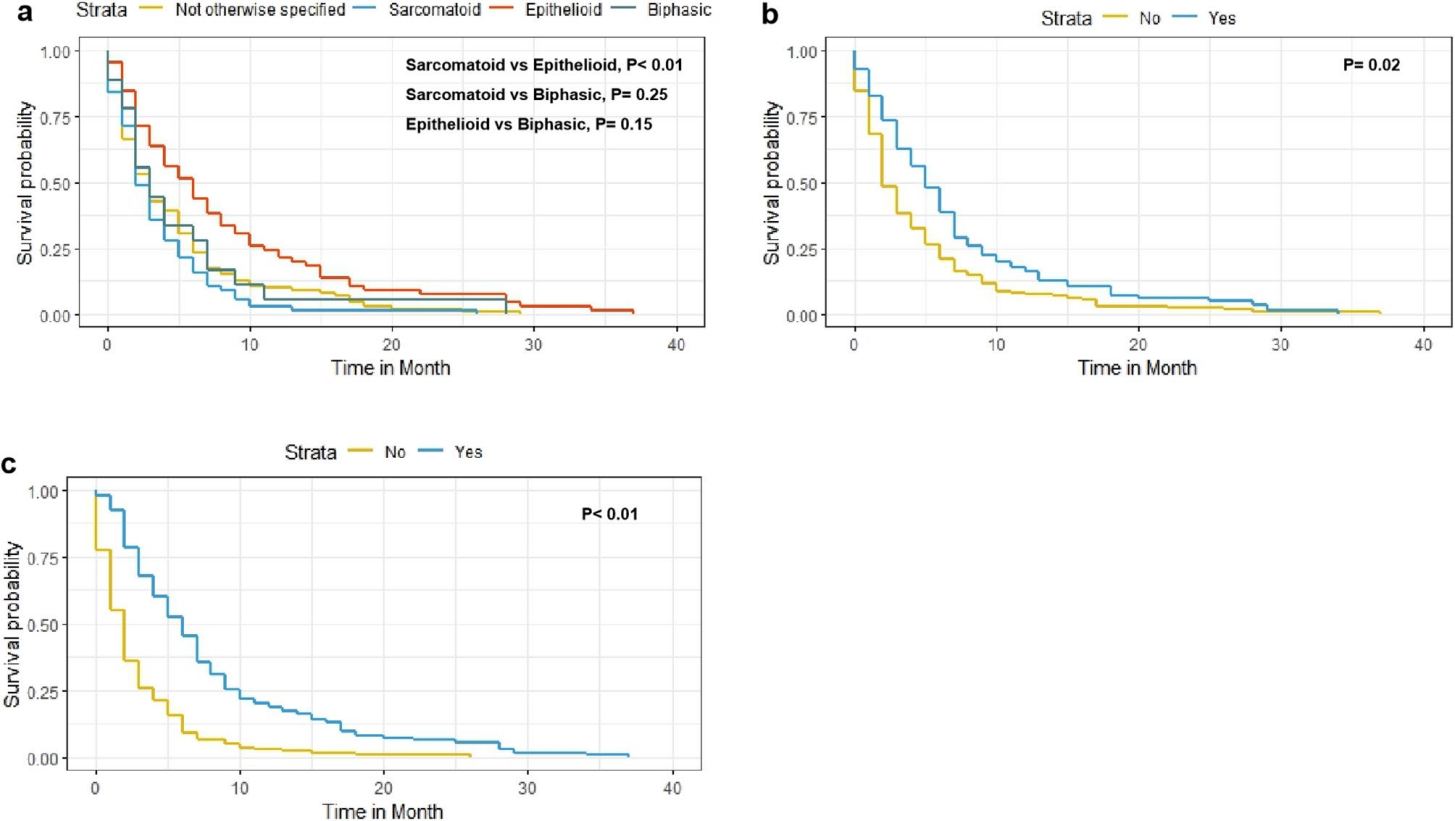
Predicted probability of overall survival by (**a**) histologict type; (**b**) radiotherapy; (**c**) chemotherapy shown using Kaplan–Meier curve.

Variables found to be significantly associated with CSS were race, age, histological type, radiotherapy, and chemotherapy were significantly associated with CSS. These variables were included in the multivariate analysis. The results showed that histological type, radiotherapy and chemotherapy were independent risk factors for CSS (Fig. 3). Combined with the results of the Kaplan–Meier survival analysis (Fig. 4), the above three independent risk factors were significantly associated with prognosis (p < 0.05).

**Figure 5:**
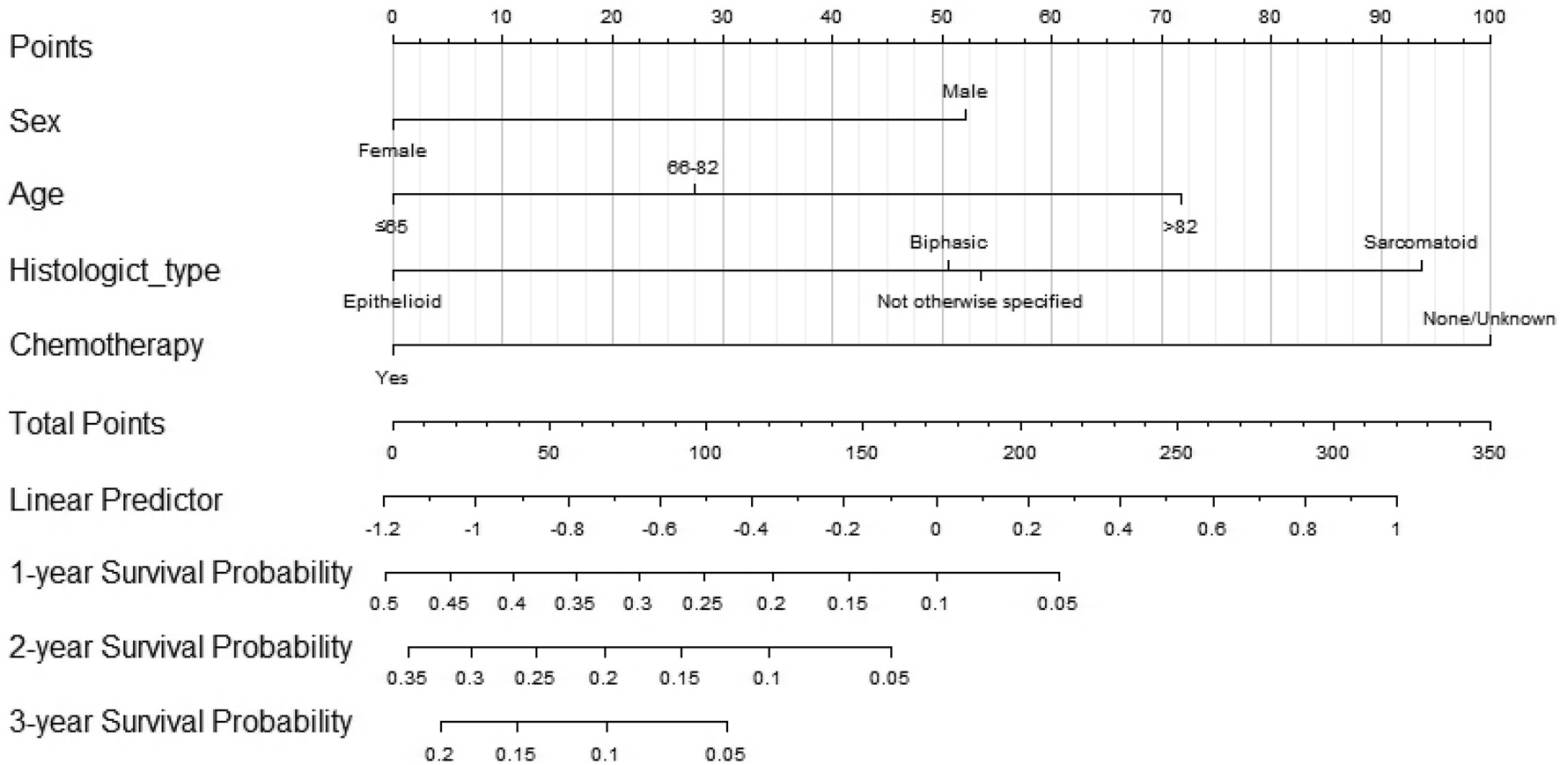
Nomograms for predicting the 1-, 2-, and 3-year overall survival of malignant mesothelioma patients with bone metastasis.

Based on the training set for the OS analysis, we constructed an OS nomogram based on four independent risk factors affecting OS (Fig. 5). According to the nomograph model, the total score was obtained by adding the scores of each factor. The risk probability corresponding to the total score is the risk probability of death of MM patients. The highest patient’s survival probability were depicted when patient's sex is female, aged 65 years old or less, with epithelioid as histological type, and has undergone chemotherapy. It can be seen that when this situation occurs, the corresponding points for each variable is 0, and the corresponding 1-year survival rate is 50%.

### Validation of nomogram

The nomogram, as a commonly used clinical prediction model, can have varying effects depending on the specific population and application scenario. Therefore, to verify that the nomogram can accurately predict outcomes in a clinical setting, it is necessary to test it on another population that was not included in the training set. Using the validation set as the internal validation data set, the discriminative degree of the model was evaluated by the area under the curve (AUC) and decision curve analysis assess the clinical utility of the model.

When we use it, we often estimate the probability of an event by making a perpendicular line. In this study, gender, age, histological type of MM, and whether chemotherapy has been performed can be calculated based on the column chart. Then, the total score of the individual can be obtained by adding the above scores, and the probability of occurrence corresponding to the total score can be estimated based on the column chart, which is the probability of death of the individual.

### Discrimination of nomogram clinical prediction models

**Figure 6:**
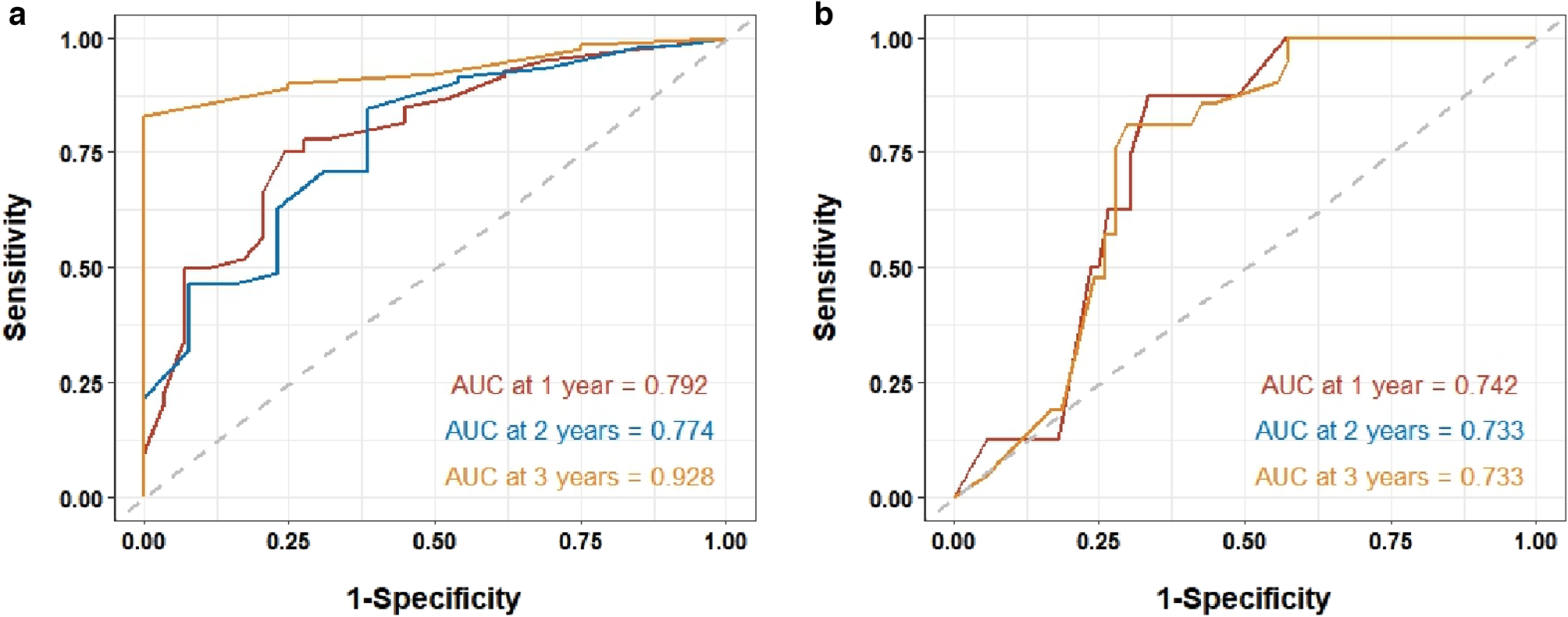
Receiver operating characteristic (ROC) curve of the nomogram. (**a**) Training set, (**b**) validation set. *AUC* area under the curve.

In the upper left corner of the ROC, sensitivity = 1, and specificity = 1. Accordingly, the closer the ROC curve is to the upper left corner of the graph, the higher the accuracy of the test. Therefore, the AUC of the ideal ROC curve is 1. As shown in Fig. 6, in our research, the 1-, 2-, and 3-year AUCs of the nomogram were 0.792, 0.774, and 0.928, and 0.742, 0.733, and 0.733 in the training and validation sets, respectively. These findings showed that that the nomogram established in this study has a good clinical application value.

### Metastasis pattern

In the MM cohort with distant metastases, 840 patients had distant metastases. The most common was single lung metastasis (325 cases), accounting for 38.69% of patients with MM with distant metastasis. Single bone (22.98%) and liver (16.55%) metastases were also more common. Single brain metastases were rarer than other single-site metastases, with only 15 (1.79%) cases. Most patients had single-organ metastasis, accounting for 80% of the cases. The most common metastases in two organs were bone and lung metastases, with 57 cases, accounting for 6.79% of the cases. Among the three-organ metastases, the most common were bone, liver, and lung metastases, accounting for 21 (2.5%) cases. The detailed results are shown in Supplementary Table S1. Supplementary Figure S3 shows a pie chart of metastatic patterns in patients with MM.

Among patients with MM with metastases, single bone, lung, and liver metastases were the main types, accounting for 78% of the total metastatic population. The log-rank test and Kaplan–Meier analysis showed that there were differences in the survival rates of these three groups, with the best prognosis for liver metastasis and the worst prognosis for bone metastasis (Supplementary Figure S4).

## Discussion

Recently, many studies have performed survival or epidemiological analyses of MM; however, the vast majority have focused on MPM. Some studies have used the SEER database to analyze MM^8–10^. In this study, we analyzed the survival of patients with MM with bone metastases using the SEER database, and in OS, 4 out of the 13 variables (sex, age, histological type, chemotherapy) were independent risk factors, and a nomogram was constructed for useful clinical application. Survival analysis showed that age, sex, histological type, and chemotherapy were independent risk factors for OS in patients with MM with bone metastases. Radiotherapy, chemotherapy and histological type were independent risk factors for CSS. However, it is important to note that histology and chemotherapy are not entirely independent risk factors as the histology influences the decisions about chemotherapy^11^. Earlier studies have shown that the majority of cases occur in men^12^, which is also reflected in our data; this may be related to occupational exposure to asbestos. Studies also have suggested that male patients had a worse prognosis compared to female patients with MM^13^. MM can occur at any age, and increasing age is an independent risk factor for MM^14^. Epithelioid malignant mesothelioma is characterized by diffuse and invasive growth of epithelioid cells from pleural surface, biphasic malignant mesothelioma is characterized by having both epithelioid and sarcomatoid components, sarcomatoid malignant mesothelioma is characterized by diffuse and infiltrative growth of spindle cells or mesenchymal cells^15^. Epithelial type tumors are the most common subtype of MM, and the non-epithelioid subtype is indicated in the guidelines for the pathological diagnosis of MM as a pathological factor associated with poor prognosis^11^, which has been confirmed in single-institution clinical studies^16^. Our study results were consistent with those of previous studies identifying prognostic risk factors.

An increasing number of studies have focused on the treatment of MM, with options including surgery, radiotherapy, chemotherapy, and immunotherapy. Whether surgery can be performed is guided by the different age, performance status, and histological type of patients^17^. Pemetrexed and cisplatin combination chemotherapy are common treatment options, but the benefits of the regimen are limited, prognosis remains poor^18^. Moreover, radiotherapy helps in controlling tumor invasion^19^. In our study, chemotherapy was an effective treatment method for prolonging OS in patients with MM with bone metastasis. Moreover, radiotherapy and chemotherapy were significantly associated with CSS.

Few studies have emphasized the role of distant metastasis in the prognostic assessment of MM, and the SEER database contains information on four types of metastatic sites. In our study, the rate of distant metastasis in MM was 12%, the top three patterns among the 15 types of metastases were single lung, single bone, and single liver metastases. Once a tumor metastasizes to the bone, it is usually incurable. The destructive consequences of bone metastasis include pathological fractures, pain, hypercalcemia, and spinal cord and nerve compression syndrome. Skeleton can also serve as a reservoir for dormant cancer cells in other organs, and bone metastasis of tumors may lead to comprehensive metastasis after prolonged dormancy. The Kaplan–Meier analysis showed that patients with liver metastasis had a batter prognosis, and that patients with bone metastasis had a worse prognosis, However, more data is needed in the future to support this conclusion. The information provided by the SEER database covers a comprehensive population, and its authenticity can be guaranteed.

However, this study has some limitations. First, this study did not conduct external validation because there was no external validation dataset. Second, the integrity of the data was difficult to guarantee owing to the many missing values. Third, in the SEER database, the treatment values of “no” and “unknown” are indistinguishable, which inevitably have effects on the analysis results.

In addition, there are only four metastatic sites in the SEER database; however, MM can also metastasize to other sites, such as the skin^20^ and oral cavity^21^. Further studies with different demographics and larger sample sizes should be conducted in the future.

## Conclusions

Age, sex, histological type, and chemotherapy are independent risk factors for OS. The OS nomogram constructed based on the above four prognostic factors has satisfactory accuracy, and its clinical utility may benefit clinical decision-making. Single-organ metastases in patients with MM are the most common, and different metastatic sites have significantly different effects on prognosis.

## Supplementary Information

Supplementary Information 1.

Supplementary Information 2.

## Data Availability

Publicly available datasets were analyzed in this study. These data are available at https://seer.cancer.gov/. The datasets supporting the conclusions of this study are included in this article.
