# Supplementary material for: Development of a prognostic nomogram for patients with malignant mesothelioma with bone metastasis
Source: Sci Rep. 2023 Jul 4;13:10789. doi: 10.1038/s41598-023-37679-9 (PMC10319832; doi:10.1038/s41598-023-37679-9)
Supplement: Supplementary file 1 — Supplementary Information 1. [file 41598_2023_37679_MOESM1_ESM.docx]

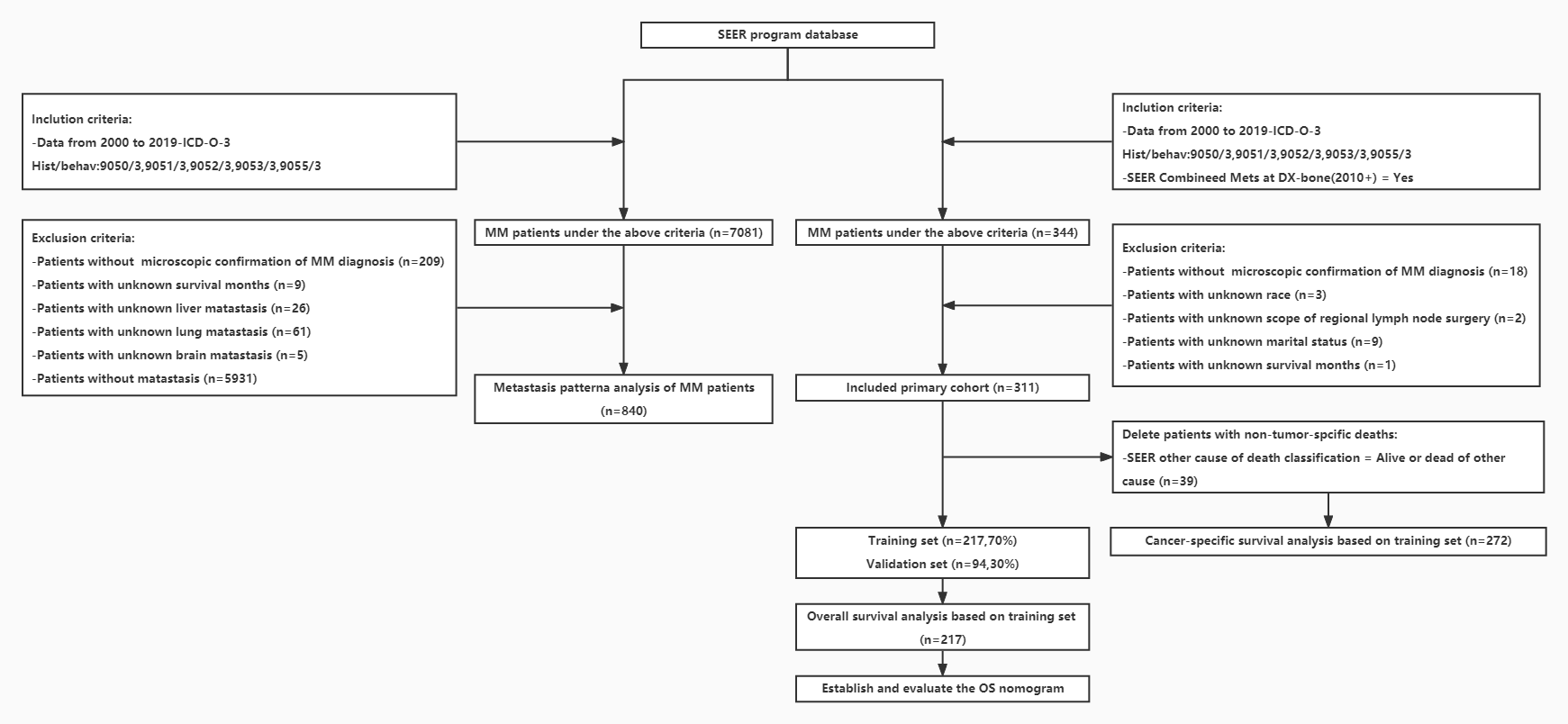


**Figure S1.** Flowchart of data selection.


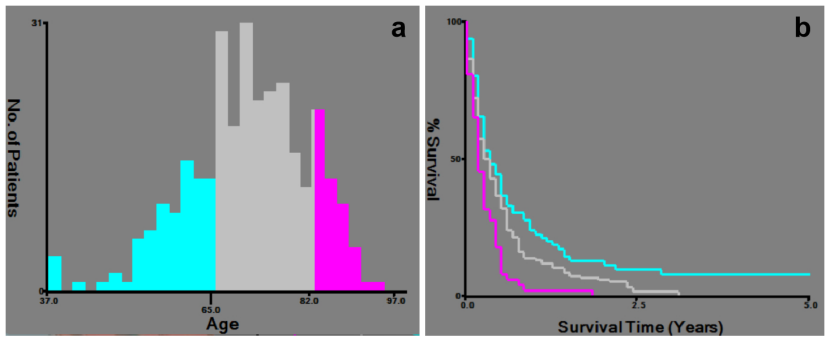


**Figure S2.** X-tile software determines optimal cutoff for age. (a) age distribution histogram and optimal cutoff value; (b) survival curves based on optimal cutoff values.

| **Parameter** | **Alive *(N,%)*** | **Dead*(N,%)*** | **ALL *(N,%)*** |
| --- | --- | --- | --- |
|  | 87(10.36) | 753(89.64) | 840 |
| Bone, brain, liver, and lung | 0(0.00) | 2(0.27) | 2(0.24) |
| Bone, liver, and lung | 0(0.00) | 21(2.79) | 21(2.50) |
| Bone, brain, and liver | 0(0.00) | 2(0.27) | 2(0.24) |
| Bone, brain, and lung | 0(0.00) | 2(0.27) | 2(0.24) |
| Brain, liver, and lung | 0(0.00) | 0(0.00) | 0(0.00) |
| Bone and liver | 0(0.00) | 28(3.72) | 28(3.33) |
| Bone and lung | 3(3.45) | 54(7.17) | 57(6.79) |
| Bone and brain | 0(0.00) | 7(0.93) | 7(0.83) |
| Liver and lung | 1(1.15) | 37(4.91) | 38(4.52) |
| Liver and brain | 0(0.00) | 1(0.13) | 1(0.12) |
| Brain and lung | 0(0.00) | 10(1.33) | 10(1.19) |
| Bone metastasis only | 15(17.24) | 178(23.64) | 193(22.98) |
| Liver metastasis only | 32(36.78) | 107(14.21) | 139(16.55) |
| Lung metastasis only | 36(41.38) | 289(38.38) | 325(38.69) |
| Brain metastasis only | 0(0.00) | 15(1.99) | 15(1.79) |

**Table S1.** Comparison of organ metastasis patterns of patients with MM.


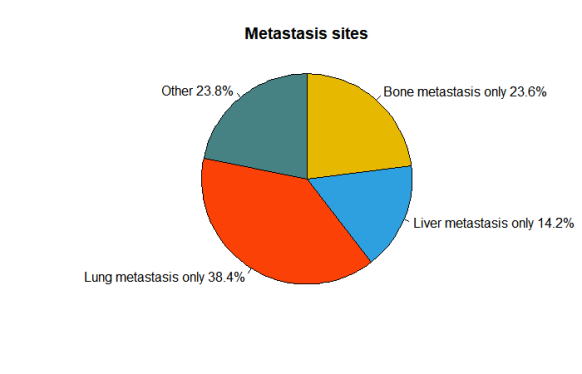


**Figure S3.** Pie chart of metastatic patterns in patients with MM.


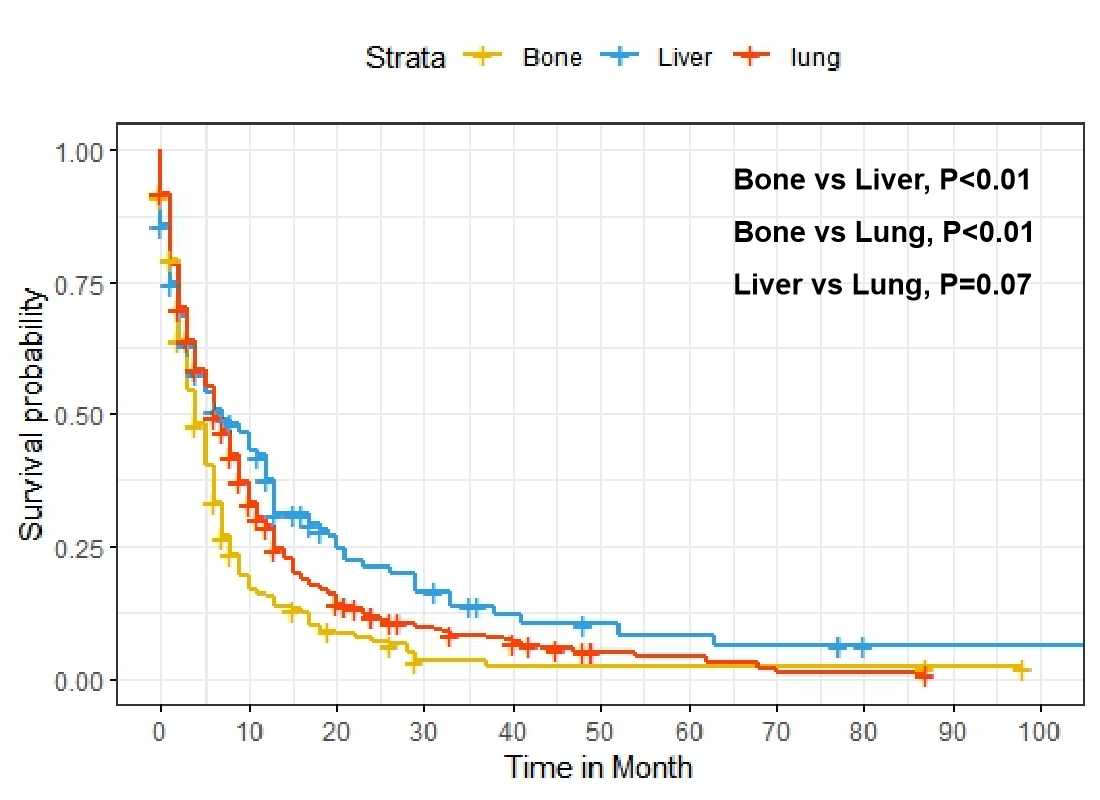


**Figure S4.** The survival difference among the different metastasis sites in patients with MM.
